# Classification and reconstruction of femoral bone defect in the revision of aseptic loosening of distal femoral endoprostheses: a 10-year multicenter retrospective analysis

**DOI:** 10.1186/s12891-022-05885-7

**Published:** 2022-10-27

**Authors:** Zi-Wei Hou, Ming Xu, Kai Zheng, Xiu-Chun Yu

**Affiliations:** 1 First Clinical Medical College, Shandong University of Traditional Chinese Medicine, Jinan, China; 2 Department of Orthopedics, The 960Th Hospital of the People’s Liberation Army, Jinan, China

**Keywords:** Revision, Aseptic loosening, Distal femoral endoprostheses, Classification, Reconstruction

## Abstract

**Objective:**

This study proposes a system for classifying the aseptic loosening of distal femoral endoprostheses and discusses reconstruction methods for revision surgery, based on different classification types.

**Methods:**

We retrospectively analyzed the data of patients who received revision surgery for aseptic loosening in distal femoral tumor endoprosthesis from January 2008 to December 2020 at 3 bone tumor treatment centers in China. Based on the patient imaging data, we proposed a classification system for the aseptic loosening of distal femoral endoprostheses and discussed its revision surgery strategy for various bone defects.

**Results:**

A total of 31 patients were included in this study, including 21 males and 10 females aged 15–75 y (average: 44.3 y). First-revision surgery was performed on 24 patients, whereas second-revision surgery was conducted on 7 patients. The 31 patients were classified into different types based on the degree of aseptic loosening: Type I, 12 patients (38.7%); Type IIa, 7 patients (22.5%); Type IIb, 7 patients (22.5%); Type III, 4 patient (12.9%); and Type IV, 1 patient (3.2%). For type I, 11 patients underwent revisions with standard longer-stem prostheses (one with the original prosthesis), and one patient had the original prosthesis plus cortical allograft strut. For type II (a and b), 10 patients underwent revisions with original prosthesis or longer-stem prosthesis and 4 patients combined with cortical allograft strut. For type III, one patient underwent revision with a longer-stem prosthesis and the other 3 patients with a custom short-stem prosthesis. For type IV, only one patient underwent revision with a custom short-stem prosthesis.

**Conclusions:**

Aseptic loosening of the distal femoral prosthesis can be divided into 4 types: type I, type II (a, b), type III and type IV. The reconstruction methods of our centers for different types of bone defects can offer some reference value in the revision of aseptic loosening.

## Introduction

Currently, tumor prosthesis is the most used technique to reconstruct bone defects after bone tumor resection [1–4]. With the continuous progress in material science and manufacturing, tumor prostheses have been widely applied because of their ability to recover keen joint function rapidly and good biological fit. Despite numerous advances in the design and production of tumor prostheses, the complication rate of tumor prostheses is considerably higher than that of ordinary knee joint prostheses [5]. Revision surgery due to complications of tumor prostheses is frequently performed [2, 6–8].

Henderson et al. [9] identified tumor endoprostheses failures and classified them into five modes: soft tissue failure (type 1), aseptic loosening (type 2), structural failure (type 3), infection (type 4), and tumor progression (type 5). Aseptic loosening is the most common failure mode in the literature. The causes and prevention of the aseptic loosening of prostheses have often been the focus of clinical research [10]. In the previous literature, the aseptic loosening rate of tumor prostheses ranged from about 3% to 16.9% [2, 4, 6, 11–22]. Literature reports indicate that the main factors affecting the aseptic loosening of prostheses are the length of resection, the type of prosthesis, the diameter of the prosthesis, and the bone-to-stem ratio [23–27]. Several studies suggest that aseptic loosening is caused by poor mechanical structure, such as the loosening or breaking of the prosthesis, are attributable to biomechanical problems [10]. These issues ultimately lead to the failure and revision of prostheses. Numerous studies have also demonstrated that aseptic loosening can be prevented by uncemented fixation [28, 29]; by using a rotating hinge structure [30] or a large-diameter stem [24]; by altering the shape of the intramedullary stem [29]; and by adding hydroxyapatite coating (HA) [30, 31].

Currently, revision for aseptic loosening of the tumor prosthesis presents a serious challenge to bone oncologists [32]. The degree of prosthesis loosening results in bone defect and residual length to varying degrees. Correct evaluation of the cortical bone of the medullary cavity and residual length are key concerns for revision surgery. No research on the assessment of aseptic loosening has been reported in the previous literature, and only few studies are conducted on the selection of reconstruction methods and surgical techniques for revision [7, 33–37]. Generally, owing to the small number of cases and large differences in location, the volume of these research evidence is insufficient. The revision strategy for tumor prostheses thus far remains inconclusive. Therefore, the classification and reconstruction of the aseptic loosening of tumor prostheses need to be proposed.

We reviewed and analyzed case data of distal femoral replacement from 3 three bone tumor diagnostic and treatment centers in China; classified the various manifestations of the aseptic loosening of tumor prostheses; and proposed the classification of aseptic loosening of femoral bone defects in distal femoral tumor prostheses; and presented our suggestions for various types of reconstruction.

## Patients and methods

### Inclusion and exclusion criteria

The inclusion criteria were as follows: (1) Tumor site at the distal femur; (2) Primary bone tumor diagnosed using clinical methods, imaging, and histopathology; (3) Reconstruction of knee joint with a tumor prosthesis; and (4) Revision surgery due to the aseptic loosening of the prosthesis. The exclusion criteria were as follows: (1) Patients with allograft prosthesis composite reconstruction in the initial operation; (2) Patients with incomplete data; and (3) Patients with tumor recurrence during and after revision surgery.

With the inclusion and exclusion criteria considered, 31 patients with distal femoral bone tumors were finally included in the study. These patients received prosthesis revision surgery at 3 bone tumor Chinese diagnostic and treatment centers from January 2008 to December 2020.

### Preoperative evolution

We reviewed the previous surgical data of each patient and recorded the preoperative clinical symptoms and function of the affected limb before the revision. Pre-revision imaging examination included a full-length positive X-ray of the lower limbs; a full-length lateral X-ray of the affected lower limb; computed tomography (CT) of the affected limb; and whole-body bone scan Emission Computerized tomography (ECT). Other imaging examinations were performed following normal procedures in accordance with the postoperative follow-up requirements for bone tumors. Preoperative X-ray, CT, and ECT examination were discussed by 1 bone and soft tissue tumor imaging expert and 2 bone tumor surgeons to determine the type of aseptic loosening of the tumor prosthesis. The preoperative examination included a routine blood test and determination of the erythrocyte sedimentation rate, C-reactive protein, and so on. Periprosthetic infection and other surgical contraindications were excluded.

### Surgical technique

Antibiotics are applied 30 min before surgery to prevent infection. During the operation, the original incision is taken to expose the prosthesis, and the loose femoral prosthesis is removed. If the prosthesis is difficult to remove, the bone cement around the prosthesis is carefully removed, followed by the femoral prosthesis. The bone cement in the medullary cavity is taken out as much as possible. The medullary cavity is opened, the inflammatory interfacial membrane tissue around the prosthesis is removed, and the blood vessels and nerves of the popliteal fossa are protected. For patients using the original prosthesis for revision, the femoral prosthesis is removed and then disinfected during surgery; the tibial prosthesis is preserved, the femoral prosthesis is again fixed with bone cement; and the polyethylene component is replaced. Patients using longer-stem prostheses for revision should decide whether to perform revision tibial prosthesis based on the fit of the femoral prosthesis and tibial prosthesis during the operation. For all revision operations, the tibial prosthesis stem is not extended; the femoral intramedullary stem of the revision prosthesis is defined as a longer stem if the length of the femoral medullary stem is more than 4 cm longer than the original prosthetic stem. For patients using a biologically fixed stem for revision, bone grafting is first performed in the femoral medullary cavity, and then the biological-stem prosthesis is implanted. Biological fixation is only used in the femoral medullary cavity, and all tibial fixation stems are fixed with bone cement. When the cortical bone of the femur has a large defect, a cortical strut allograft is used outside the femur.

### Follow-up

This study required a follow-up by revision surgeons on all patients. The follow-up was completed by outpatient review once every 3 months for 2 y after surgery, once every 6 months during 2–5 y, and once annually after 5 y. The follow-up content mainly included the presence of pain in the affected limb and new symptoms of discomfort, the Musculoskeletal Tumor Society (MSTS) functional score to evaluate the function of the affected limb [38], the X-ray examination of the surgical site, and so on. Other routine examinations were conducted in accordance with the requirements for follow-up after the primary tumor surgery.

### Classification of aseptic loosening

**Fig. 1:**
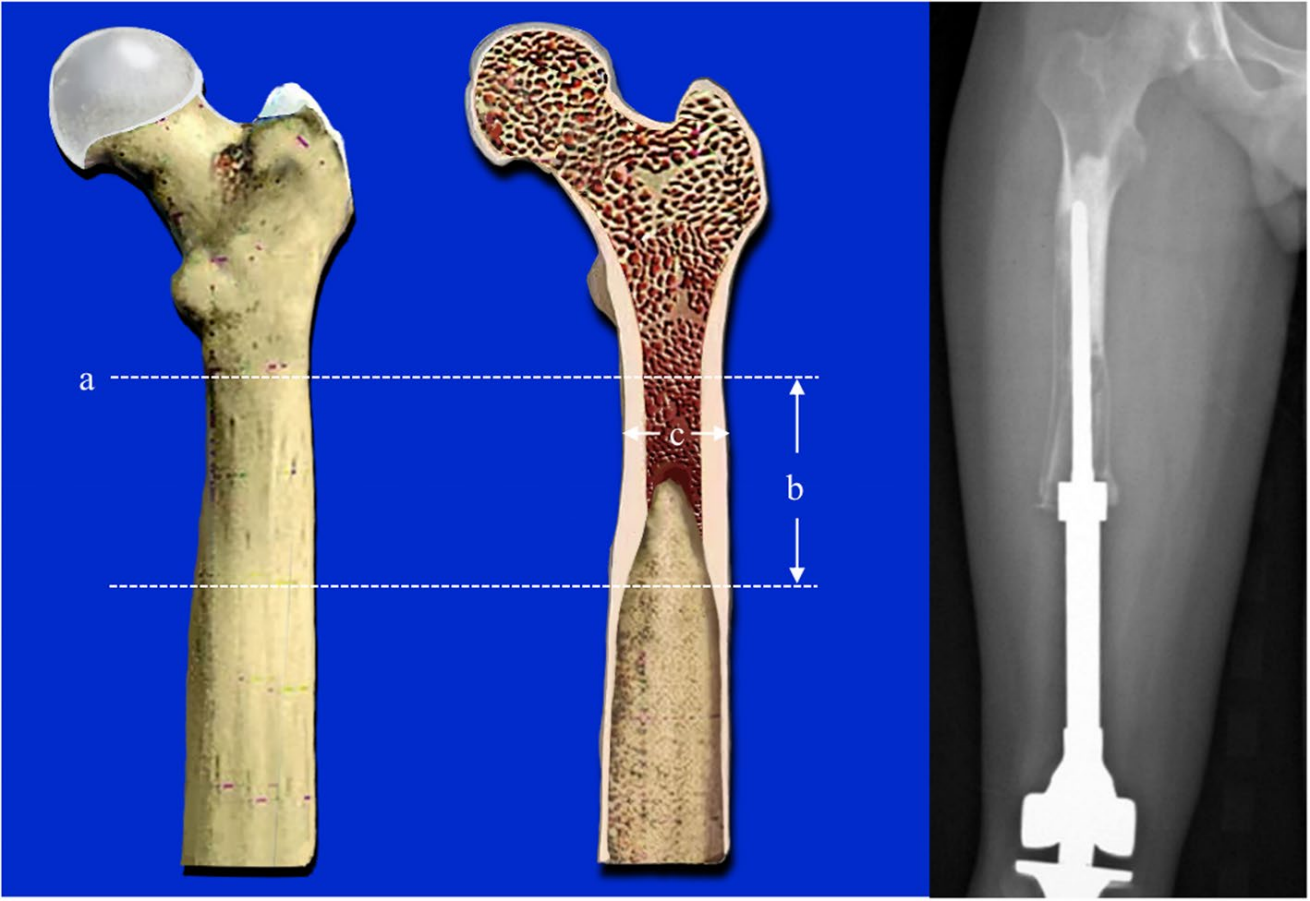
The prosthesis has no displacement, and the isthmus is intact. **a**: the highest horizontal line of the isthmus. **b**: the length of can be used for fixation ≥ 5 cm / 2 times the diameter of the medullary cavity. **c**: the diameter of the medullary cavity

Type I: The prosthesis has no displacement, and the length of the medullary cavity below the highest horizontal line of the isthmus can be used for fixation ≥ 5 cm / 2 times the diameter of the medullary cavity (Fig. 1).

Type II: The prosthesis is displaced, and the length of the medullary cavity below the highest horizontal line of the isthmus can be used for fixation ≥ 5 cm / 2 times the diameter of the medullary cavity.

**Fig. 2:**
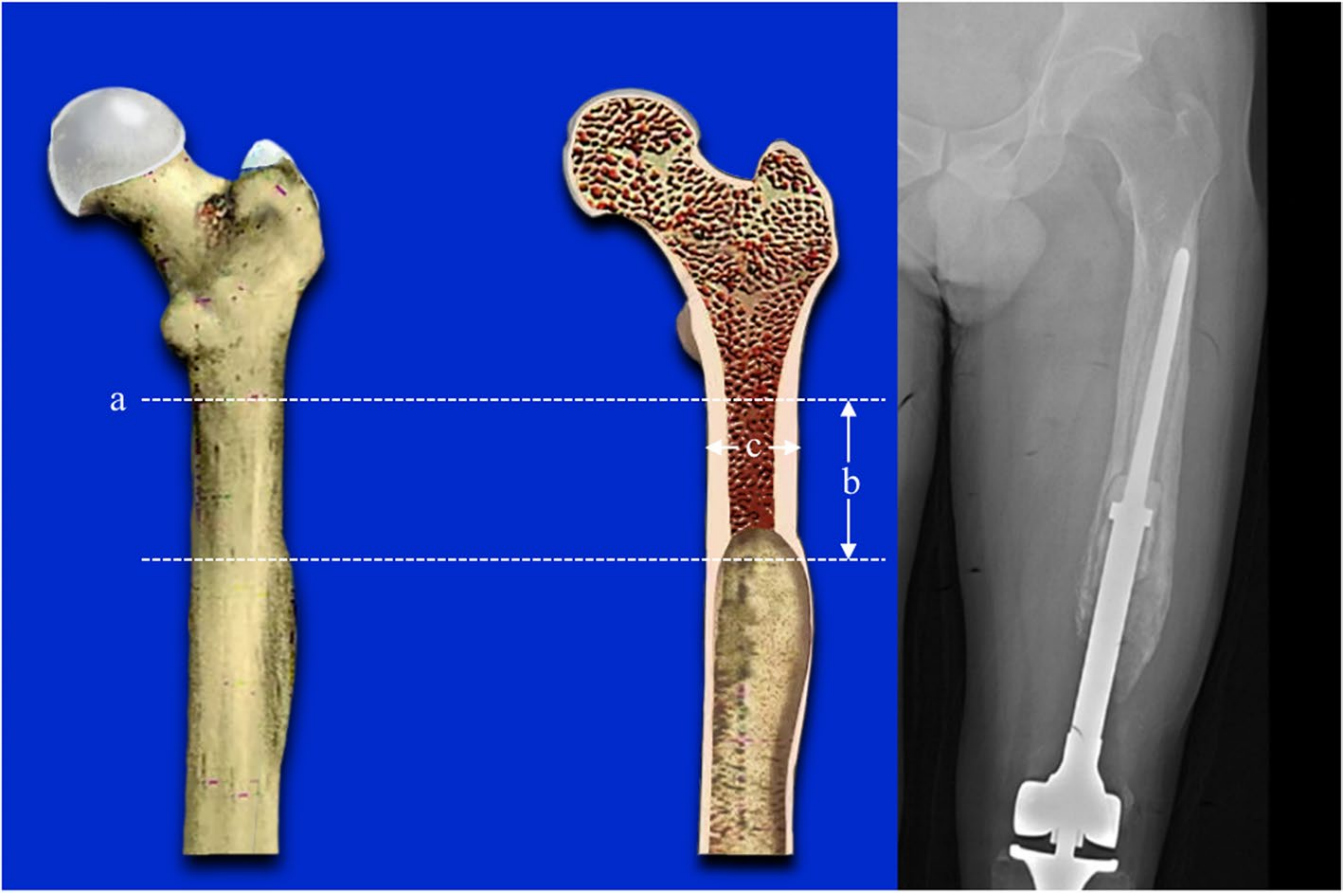
The prosthesis is displaced. The femoral cortical bone bulges but the femoral medullary canal is intact. **a**: the highest horizontal line of the isthmus. **b**: the length of can be used for fixation ≥ 5 cm / 2 times the diameter of the medullary cavity. **c**: the diameter of the medullary cavity

Type II a: The femoral cortical bone bulges but the femoral medullary canal is intact (Fig. 2).

**Fig. 3:**
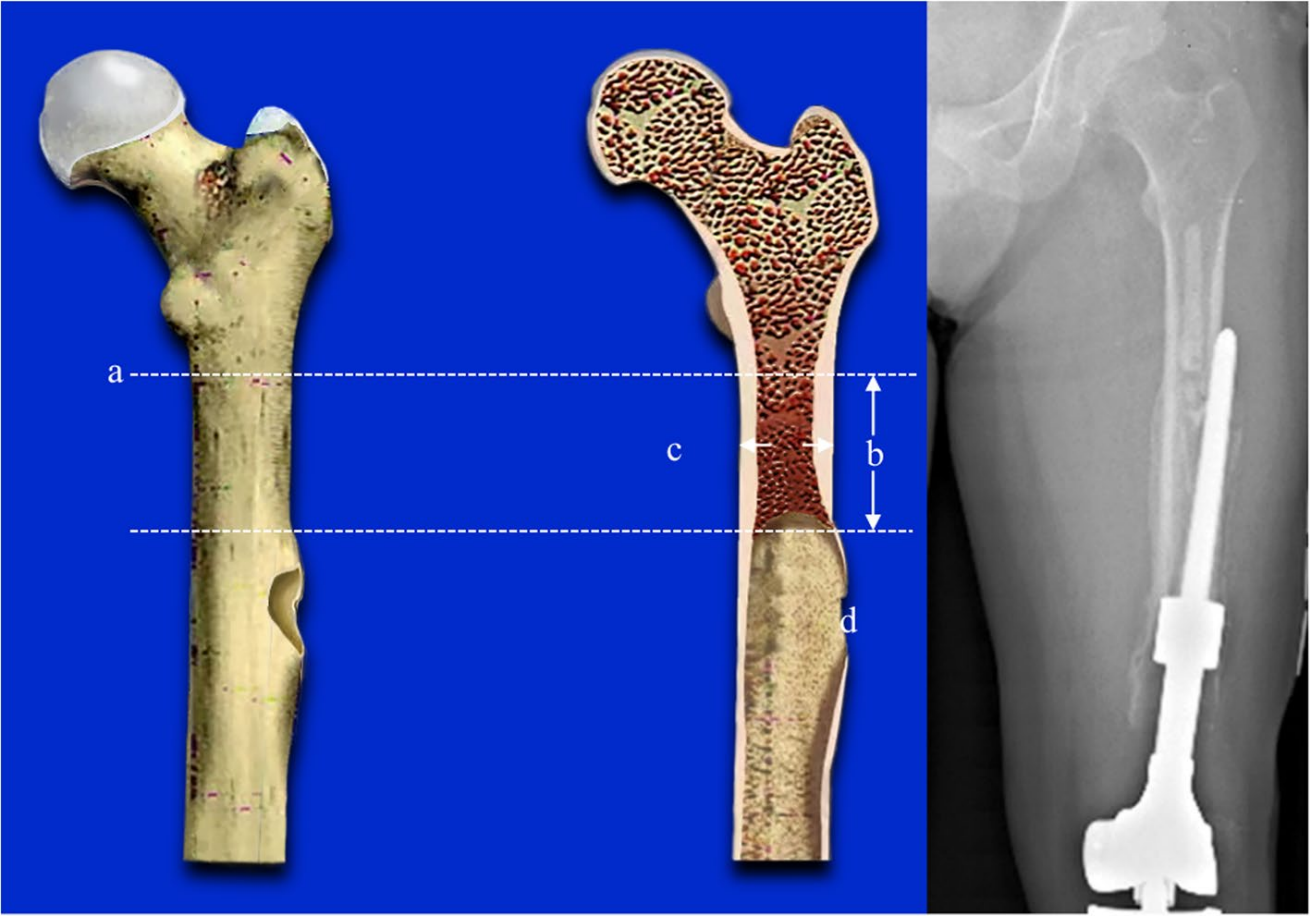
The femoral cortical bone bulges and the prosthesis penetrate the cortical bone. **a**: the highest horizontal line of the isthmus. **b**: the length of can be used for fixation ≥ 5 cm / 2 times the diameter of the medullary cavity. **c**: the diameter of the medullary cavity. d: the prosthesis penetrates the cortical bone

Type II b: The femoral cortical bone bulges and the prosthesis penetrates the cortical bone (Fig. 3).

**Fig. 4:**
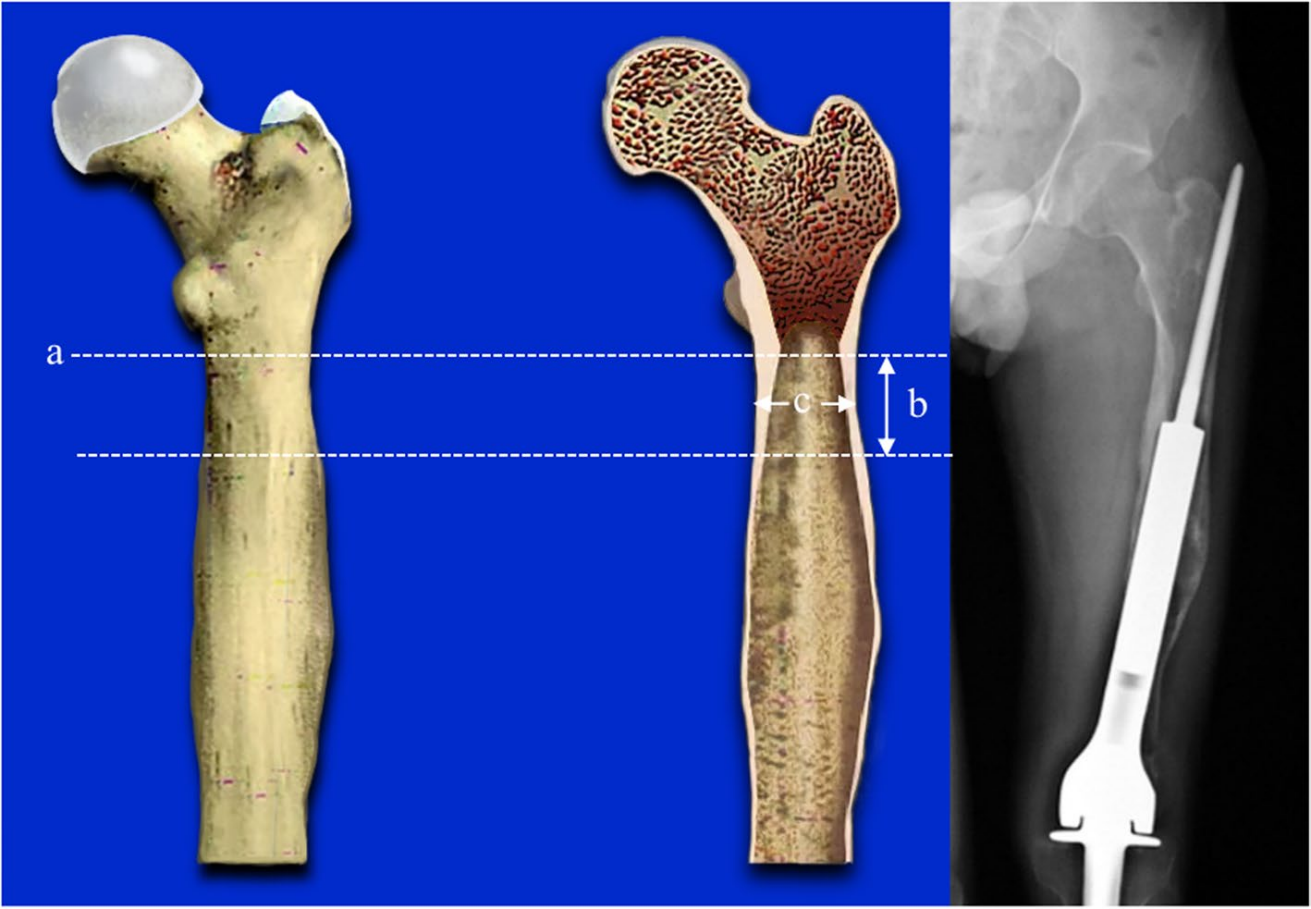
The bone defect involved the isthmus. **a**: the highest horizontal line of the isthmus. **b**: the length of can be used for fixation < 5 cm / 2 times the diameter of the medullary cavity. **c**: the diameter of the medullary cavity

Type III: The length of the medullary cavity below the highest horizontal line of the isthmus can be used for fixation < 5 cm / 2 times the diameter of the medullary cavity (Fig. 4).

**Fig. 5:**
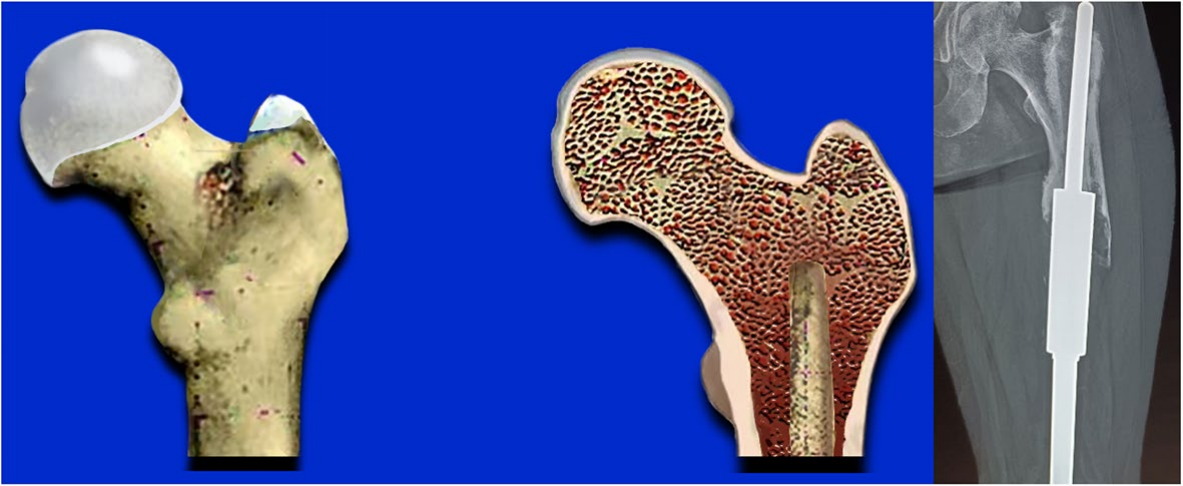
Isthmus femoral medullary cavity has been lost, and/or bone defects involving the metaphysis

Type IV: Isthmus femoral medullary cavity has been lost, and/or bone defects involving the metaphysis (Fig. 5).

### Statistical analysis

This research is a retrospective study. Owing to the rare occurrence of clinical cases and small sample size, no statistical comparison and analysis were conducted. All data presented are descriptive.

## Results

A total of 31 patients, which consist of 21 males and 10 females aged 15–75 y (average age: 44.3 y), were included in this study. Of this number, 24 patients underwent a first-revision surgery, and the remaining 7 patients received a second-revision surgery. The patient counts with their corresponding types of aseptic loosening (of the distal femoral tumor prosthesis) were as follows: Type I, 12 patients; Type IIa, 7 patients; Type IIb, 7 patients; Type III, 4 patients; and Type IV, 1 patient (Table 1). Ultimately, 26 patients underwent MSTS functional score evaluation.

**Table 1:** Proportion of different type

Type	*N* (%)
I	12(38.7%)
IIa	7(22.5%)
IIb	7(22.5%)
III	4(12.9%)
IV	1(3.2%)

**Fig. 6:**
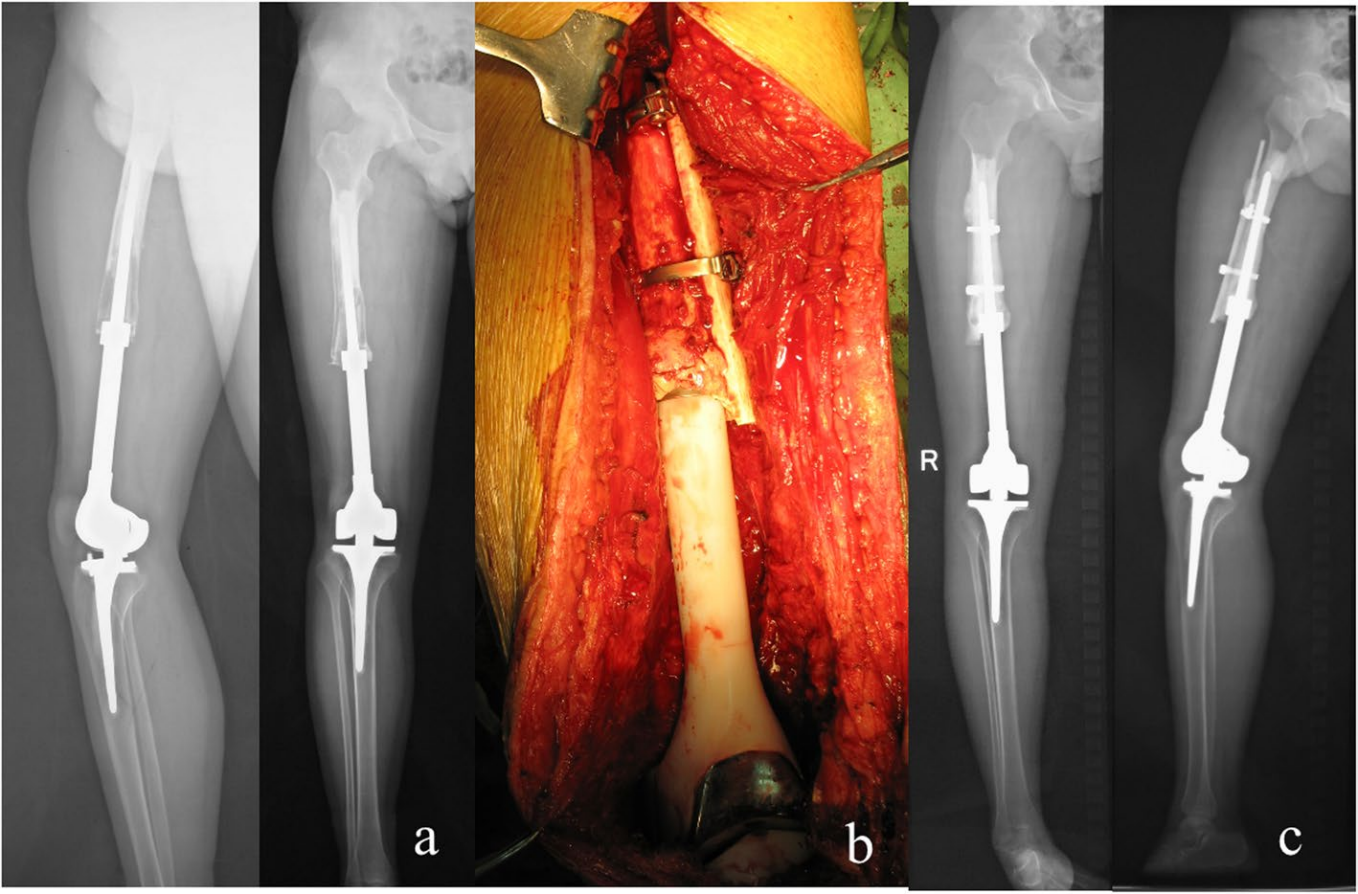
**a**: The prosthesis was not displaced before surgery, the isthmus was intact, and the bone cortex around the prosthesis was absorbed, belonging to type I. **b**: The original prosthesis and cortical strut allograft were used for revision during the operation. **c**: The prosthesis was in a good position during 5 years of follow-up

The cases classified as Type I included 12 patients aged 15–64 (average 40) y; 4 patients had osteosarcoma, 5 patients were diagnosed with giant cell tumor of bone, and 3 patients had other tumors (1 chondrosarcoma, 1 desmoid-type fibromatosis, and 1 malignant fibrous histiocytoma). 10 patients were the first revision, and 2 patients were the second revision. Nine patients used a rotating hinge prosthesis in their primary replacement and three had a fixed hinge prosthesis. The prosthesis had been in place for 1–15 y, with an average time of 6.25 y. 11 patients underwent revisions with standard longer-stem prostheses (one with the original prosthesis), and one patient had the original prosthesis plus allogeneic cortical bone (Case7, Fig. 6). One patient with chondrosarcoma died of lung metastases 2 and 1 y after the operation. One patient survived with lung metastasis after the operation. One patient with the second revision underwent amputation due to postoperative infection, and the other patient with the second revision underwent bone lengthening and knee fusion due to severe limb shortening. All patients were followed up for 1–10 y, and the average MSTS functional score was 27.7 (26–29) (Table 2, 3, 4).

**Table 2:** Details of diagnosis and primary replacement in 31 patients

Case	Gender	Age(yrs)	Site	Pathological diagnosis	Primary replacement	Survival time of endoprosthesis (yrs)	Type
1	male	49	R	OS	RHP	2	I
2	male	22	L	OS	RHP	1	I
3	male	33	L	GCT	RHP	4	I
4	male	15	R	OS	RHP	4	I
5	male	64	R	GCT	RHP	3	I
6	male	59	R	GCT	FHP	11	I
7	male	17	R	OS	RHP	5	I
8	male	45	R	CS	FHP	1	I
9	female	46	R	DF	FHP	13	I
10	female	49	R	GCT	RHP	15	I
11	female	33	R	GCT	RHP	13	I
12	male	49	L	MFH	RHP	3	I
13	male	40	L	GCT	RHP	11	IIa
14	male	37	L	GCT	FHP	14	IIa
15	female	25	L	OS	RHP	8	IIa
16	male	31	L	OS	RHP	4	IIa
17	female	38	R	OS	RHP	11	IIa
18	female	64	R	GCT	FHP	20	IIa
19	male	39	R	GCT	RHP	7	IIa
20	male	58	L	MPNST	RHP	3	IIb
21	male	40	L	GCT	RHP	11	IIb
22	female	38	R	GCT	FHP	10	IIb
23	male	50	L	GCT	RHP	14	IIb
24	male	53	L	GCT	RHP	9	IIb
25	male	54	L	GCT	RHP	12	IIb
26	female	60	L	GCT	FHP	27	IIb
27	male	31	L	OS	FHP	14	III
28	male	48	L	GCT	RHP	13	III
29	female	75	L	GCT	RHP	14	III
30	male	58	L	GCT	RHP	7	III
31	female	56	L	OS	RHP	7	IV

*R* Right, *L* Left, *OS* Osteosarcoma, *GCT* Giant cell tumor, *CS* Chondrosarcoma, *DF* Desmoid-type Fibromatosis, *MHF* Malignant fibrous histiocytoma, *MPNST* Malignant peripheral nerve sheath tumor, *FHP* Fixed hinge prosthesis, *RHP* Rotating hinge prosthesis

**Table 3:** Details of revisions and follow-up in 31 patients

Case	Revision	Reconstruction	Follow-up since revision surgery(yrs)	Length of Shortening (cm)	Outcome/Complication(s)	MSTS scores
1	First	OP	2	-	Died of pulmonary metastasis	-
2	First	Longer-stem RHP	2	-	Survival with pulmonary metastasis	29
3	First	Longer-stem RHP	4	-	DFS	29
4	First	Longer-stem RHP	3	-	DFS	27
5	First	Longer-stem RHP	4	1	DFS	29
6	Second	Longer-stem RHP	11	-	DFS/Infection	-
7	Second	OP + CSA	5	-	DFS/Fracture	-
8	First	Longer-stem RHP	1	-	Dead	-
9	First	Longer-stem FHP	10	-	DFS	-
10	First	Longer-stem RHP	2	-	DFS	27
11	First	Longer-stem RHP	2	2	DFS	26
12	First	Longer-stem RHP	4	3	DFS	27
13	Second	Longer-stem RHP	2	8	DFS	22
14	First	Longer-stem RHP + CSA	10	2	DFS	27
15	First	Longer-stem RHP	8	2	DFS	27
16	First	Longer-stem RHP	8	1	DFS	27
17	First	OP + CSA	5	2	DFS	27
18	First	Longer-stem RHP	4	3	DFS	25
19	First	Longer-stem RHP	4	-	DFS	26
20	First	OP + CSA	4	-	DFS	25
21	First	Longer-stem RHP	3	2	DFS	27
22	First	OP	16	5	DFS	26
23	First	Longer-stem RHP + CSA	2	3	DFS	27
24	First	Longer and larger stem RHP	2	2	DFS	27
25	First	Longer-stem RHP	4	-	DFS	26
26	First	Longer-stem RHP	4	6	DFS	26
27	First	Longer-stem FHP	10	9	DFS	20
28	Second	Custom Short Stem RHP	1	-	DFS	20
29	Second	Custom Short Stem RHP	1	-	DFS	21
30	Second	Custom Short Stem RHP	1	-	DFS	20
31	Second	Custom Short Stem RHP	1	1.5	DFS	22

*OP* Original prosthesis, *FHP* Fixed hinge prosthesis, *RHP* Rotating hinge prosthesis, *CSA* Cortical strut allograft, *DFS* Disease-free survival

**Table 4:** Summary of different types of diagnosis and treatment

Variable		I	IIa	IIb	III	IV
Average age (yrs)		40(15 ~ 64)	39(25 ~ 64)	50(38 ~ 60)	53(31 ~ 75)	56
Type of tumor	OS	4	3	-	1	1
	GCT	5	4	6	3	-
	CS	1	-	-	-	-
	DF	1	-	-	-	-
	MFH	1	-	-	-	-
	MPNST	-	-	1	-	-
Prosthesis before revision	First/Second revision	10/2	6/1	7/0	1/3	0/1
	Fixed/Rotating	3/9	2/5	2/5	1/3	1/0
	Prosthetic survival time	6.25(1 ~ 15)	11(4 ~ 20)	11(3 ~ 27)	12(7 ~ 15)	7
Methods of revision	OP/Longer stem	11	5	5	1	-
	Allograft + Longer-stem/OP	1	2	2	-	-
	Custom short stem	-	-	-	3	1
MSTS score		27.7(26 ~ 29)	25.8(22 ~ 27)	26.2(25 ~ 27)	20.2(20 ~ 21)	20

*OS* Osteosarcoma, *GCT* Giant cell tumor, *CS* Chondrosarcoma, *DF* Desmoid-type Fibromatosis, *MHF* Malignant fibrous histiocytoma, *MPNST* Malignant peripheral nerve sheath tumor, *OP* Original prosthesis

Under Type IIa were 7 patients aged 25–64 y; 4 of the patients were diagnosed with giant cell tumor of the bone, and 3 patients had osteosarcoma. One patient underwent a second revision, and the other 6 patients underwent a first-revision surgery. Before the revision surgery, 2 patients used the fixed hinge prosthesis, and 5 patients used the rotating hinge prosthesis. The prosthesis had been in place for 4–20 y, with an average time of 11 y. One patient used the original prosthesis plus cortical allograft strut for revision, and 6 patients used the longer-stem rotating hinge prosthesis (1 plus cortical allograft strut) for revision. All patients survived, tumor-free. The postoperative follow-up was 2–10 y. The patients’ limbs were shortened by 1–8 cm, and the average MSTS score was 25.8(22 ~ 27) (Table 2, 3, 4).

**Fig. 7:**
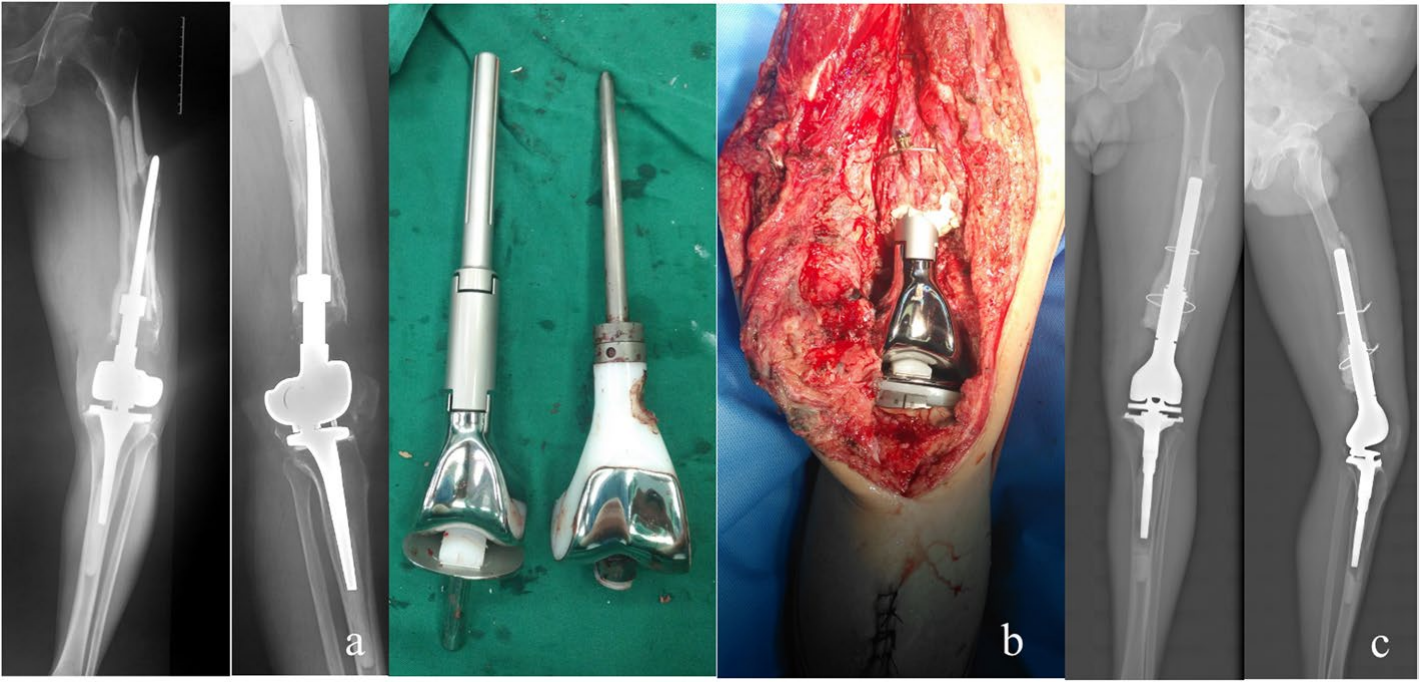
**a**: The prosthesis was displaced before surgery, the isthmus was intact, and the prosthesis broke through the cortical bone, which belonged to type IIb. **b**: A longer and thicker stem prosthesis was used for revision during the surgery. **c**: The prosthesis was in a good position 2 years after surgery

Type II b consisted of 7 patients aged 38–60; 6 patients were diagnosed with giant cell tumor of the bone, and 1 patient had a malignant peripheral nerve sheath tumor. All patients underwent primary revision. Fixed hinge prostheses were used in 2 patients, and rotating hinge prostheses were used in 5 patients before revision surgery. These prostheses had been in place for 3–27 y, with an average time of 11 y. Revisions were conducted using the original prosthesis in 1 patient; the original prosthesis plus cortical allograft strut in 1 patient; the longer-stem prosthesis plus cortical allograft strut in 1 patient; and longer-stem rotating hinge prosthesis in 4 patients (Case 24, Fig. 7). All patients survived, tumor-free, and were followed up for 2–16 y postoperatively. The limbs of the patients were shortened by 2–6 cm, and their average MSTS score was 26.2(25 ~ 27) (Table 2, 3, 4).

**Fig. 8:**
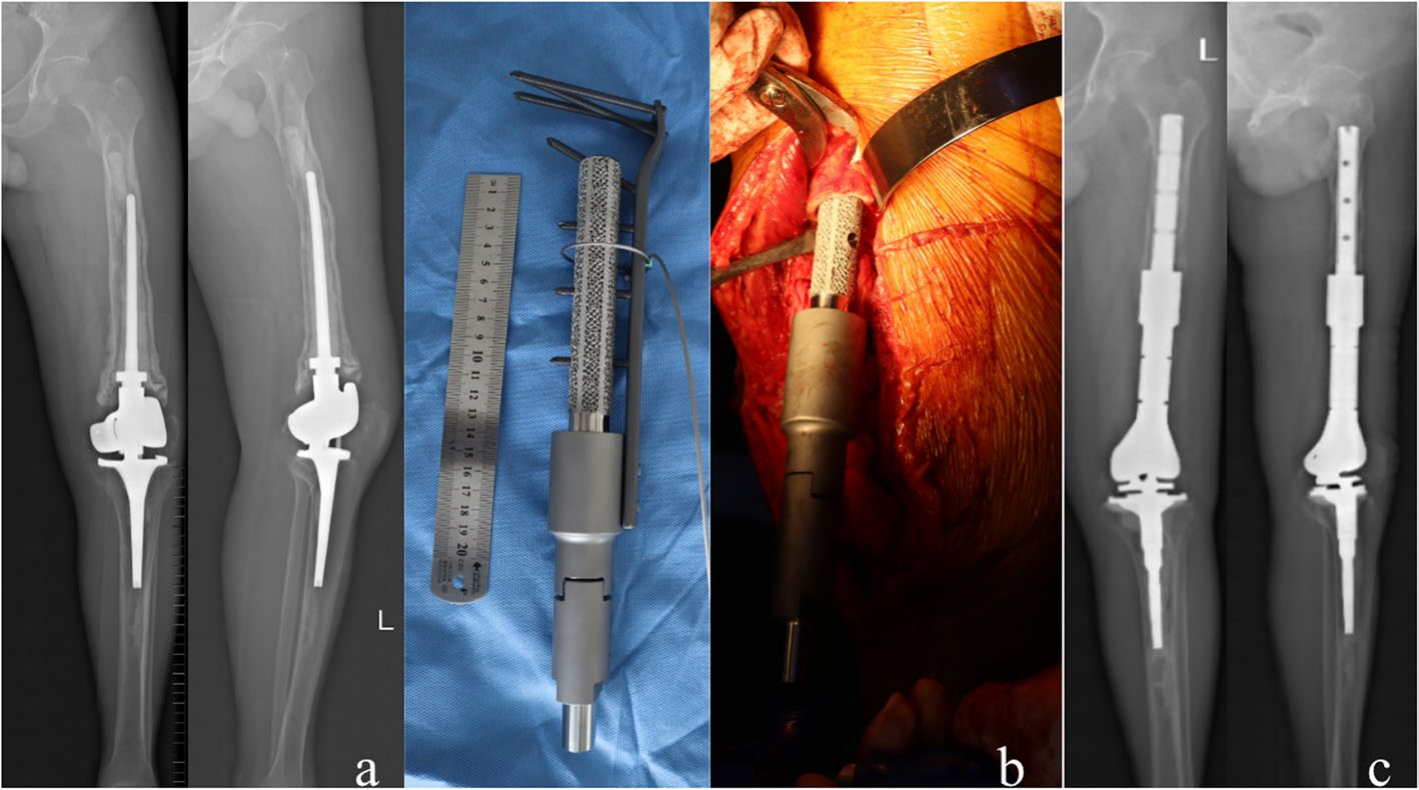
**a**: The femoral canal is widened due to preoperative bone absorption around the prosthesis, and the isthmus can be used to fix the length < 5 cm, which belongs to type III. **b**: A custom short stem prosthesis was used for revision during the operation. **c**: The prosthesis was in a good position 2 years after surgery

The case under Type III was 4 patients aged 31–75 y, 3 of the patients were diagnosed with giant cell tumor of the bone, and one patient had osteosarcoma. One patient with a fixed hinge prosthesis underwent the first revision, and the other 3 patients with rotating hinge prostheses underwent the second revision. The patient who underwent the first revision used a longer-stem prosthesis, and the other 3 patients who had the second revision used a custom short-stem prosthesis (Case28, Fig. 8). All patients survived, tumor-free, and were followed up for 1–10 y postoperatively. The limbs of the patients were shortened by 1.5–9 cm, and their average MSTS score was20.2(20 ~ 21) (Table 2, 3, 4).

**Fig. 9:**
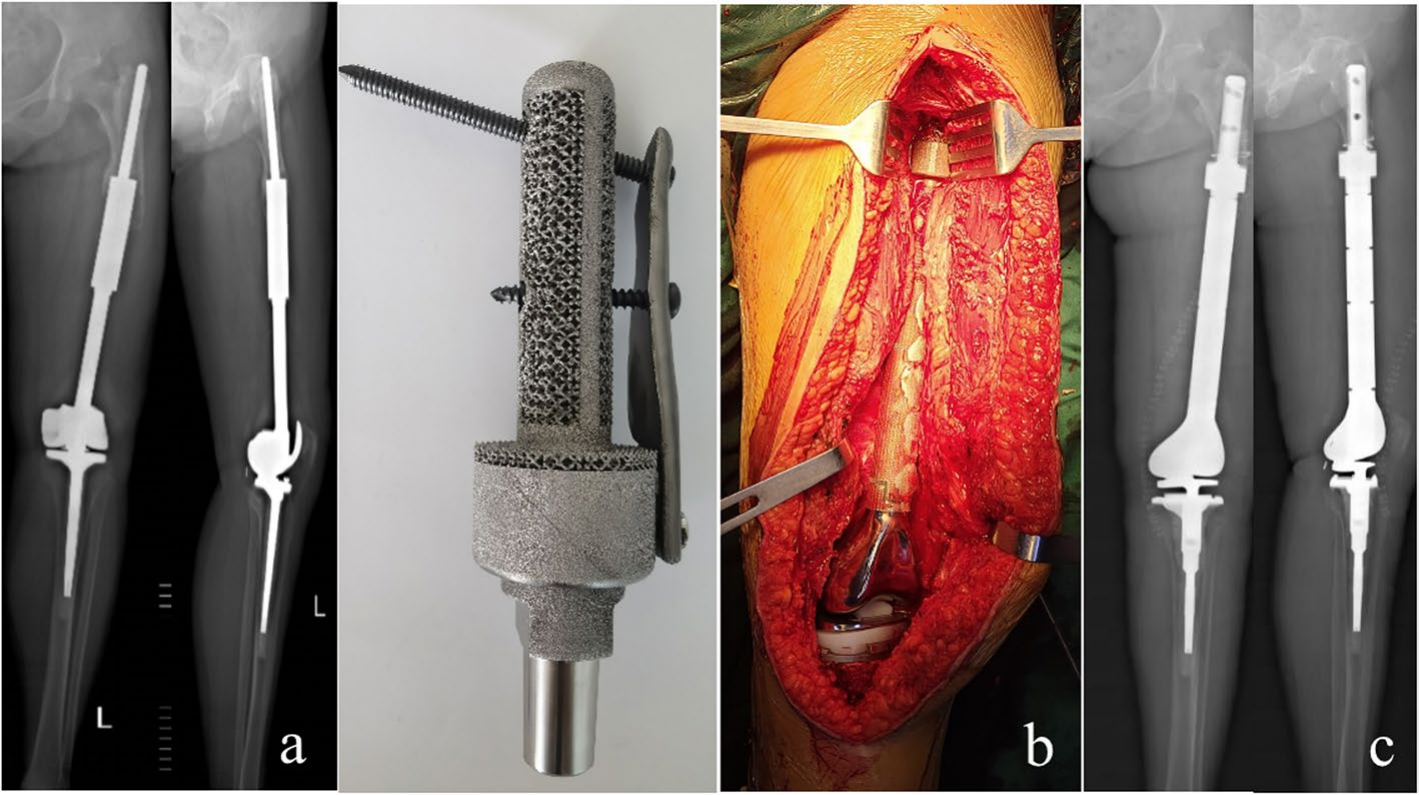
**a**: Preoperative imaging showed that the medullary cavity of the isthmus had been lost, and the bone defect involved the metaphysis, which belonged to type IV. **b**: Revision was performed with a custom short stem prosthesis during the operation. **c**: The prosthesis was in good condition at 1-year follow-up

Type IV was a 56-year-old female patient with osteosarcoma who underwent a primary revision of a rotating hinge prosthesis 7 years ago and a second revision with a custom short-stem prosthesis (Case 31, Fig. 9). After 1-year follow-up postoperatively, the limb was shortened by 1.5 cm, and the MSTS score was 20 (Table 2, 3, 4).

## Discussion

Limb salvage surgery is a major surgical technique for a malignant bone tumor in the extremities [39, 40]. Currently, prosthetic reconstruction is widely used in clinical practice after tumor resection. Despite continuous improvement in the material and design of the prosthesis, the implantation failure rate of tumor prostheses remains higher than that of nontumor prostheses. Previous literature reviews showed that the 5-year survival rate of patients with knee tumor prostheses ranged from 57 to 93%, and the 10-year survival rate of the same was 50%–88% [1, 4, 9, 13]. Aseptic loosening is a common prosthesis failure mode [9], and its occurrence may be related to age, tumor resection length, prosthetic stem size, and the biomechanical instability [10, 23–27].

Distal femoral replacement (DFR) comprises a large proportion of prosthetic replacement and is prone to aseptic loosening and revision surgery [5, 10, 41]. Aseptic loosening of DFR prostheses often results in bone absorption to varying degrees and even destruction. Consequently, residual bone mass is lost, and the proximal femur is shortened, inevitably causing challenges to revision surgery [32, 42]. Therefore, correct understanding and evaluation of bone defects after aseptic loosening are important prerequisites for revision surgery.

We reviewed the data of 31 patients with aseptic loosening and revision after DFR from 3 bone tumor diagnostic and treatment centers in China. The aseptic loosening of the distal femoral tumor prosthesis was classified by 1 expert in bone and soft tissue tumor imaging and 2 bone tumor surgeons. On the basis of the aforementioned classification, the differences in the performance of prosthetic displacement, the integrity of the isthmus, femoral residual bone mass, and length on X-ray, a new classification of bone defects was proposed. Under the new classification system, the defects were divided into 4 types. We also summarized the revision techniques performed on 31 patients by different types and tried to suggest specific revisions. We hope that the proposed classification of aseptic loosening is expected to elucidate the aseptic loosening of distal femoral tumor prostheses, as well as provide our revision strategy for DFR.

Aseptic loosening under Type I usually occurs shortly after surgery. The literature shows that tumor prosthesis failure has the highest incidence rate in the early stages, comprising 69% of the total within 5 y [4]. 12 patients with Type I loosening underwent revision surgery, with an average prosthetic survival time of 6.25 y before revision; of this number, 4 patients had osteosarcoma (Table 2, 3, 4). Early loosening may be attributed to perioperative chemotherapy and extensive resection boundaries. Aseptic loosening under Type I has no displacement, and the bone structure around the prosthesis is normal; the revision is relatively simple and similar to the initial prosthetic replacement. Although the bone around the prosthesis becomes thinner, the bone inclusivity is still complete, providing a good bone implantation environment for the prosthesis. Moreover, the bone structure in the proximal femur has a certain normal length, which can be revised by original or longer-stem prosthesis. In Type I aseptic loosening, the prosthesis has no displacement, the bone defect caused by loosening is less, and the amount of bone available for fixation in the proximal femur is sufficient. Therefore, the use of original prostheses or longer-stem prostheses for reconstruction should be considered in most revision surgery of type I. In addition, the use of cortical strut allograft may also be considered by a small number of patients with severe bone loss but no displacement of the prosthesis.

The prosthesis with Type IIa loosening showed displacement and absorption of bone, but its integrity still existed. Under this type, 7 patients underwent revision surgery, with an average prosthetic survival time of 8 y, and the affected limbs had a certain degree of shortening (Table 2, 3, 4). The limb shortening that can be corrected by revision surgery is limited, which should be communicated to the patient before the operation. The prosthesis with Type IIb loosening showed a large displacement, and the prosthesis stem pierced the broken bone cortex. Seven patients were reported to show this type of bone defect at 11 y postoperatively. This type is usually accompanied by a bad force line and limb shortening. Our data showed that revision of type II followed the same strategy as type I, with original or longer-stem prosthesis in 10 patients and a combination of allograft in 4 patients (Table 2, 3, 4). The allograft can effectively help to fill the bone defect and provide partial support. A longer stem can avoid the stress concentration point of the original prosthesis, as well as reduce the possibility of the stem penetrating the cortex of bone once again. Therefore, the use of longer-stem prostheses plus allograft for reconstruction should be considered in most revision surgery of type II.

In Type III aseptic loosening, the length of the medullary cavity below the highest horizontal line of the isthmus can be used for fixation < 5 cm (or less than 2 times the diameter of the medullary cavity). This type of loosening often occurs when long segmental bone defects are reconstructed with prostheses after large segmental resection of distal femoral tumors. The reason is that the length of the isthmus for fixation is too short, the contact surface between the prosthesis stem and bone is reduced, and the holding force of the stem is inadequate. Four patients were classified under Type III loosening in our study, and the custom short stem prosthesis (with porous structure) was used in their revisions (Table 4). The revision of type III loosening presents a challenge; thus, a good implantation environment is necessary for the prosthesis. Currently, the commonly used methods reported in the literature are the telescope tube-like allograft prosthesis [43–45] and the Compress® Compliant Pre-stress (CPS) prosthesis [46–48]. The telescope technology was first proposed by Healey et al. [43] and succeeded allogeneic bone grafts used in reconstruction and revision [49, 50]. With telescope technology, the grafted bone and the host bone are overlapped to maximize the surface contact between the host bone and the allogeneic bone to realize a stable fixation of the distal end of the prosthesis [43]. Hindiskere et al. [45] confirmed the effectiveness of telescope technology. In 14 patients with telescope bone allograft, the bone healing rate was 100%, and the MSTS score was 27 at the final follow-up. Another commonly used technique reported in the literature is compressive osseointegration, which uses the axial pressure between the implant and the bone surface for initial implant fixation [51]. With this approach, the large prosthesis can be fixed to a considerably short backbone segment. In addition, compressive osseointegration can effectively avoid stress shielding, which can induce bone hypertrophy and inward growth at the interface between bone and prosthesis [48, 51, 52]. CPS prosthesis can be used to repair distal femoral prosthesis with a short femoral stump and numerous bone defects [46, 47].

In Type IV loosening, the medullary cavity of the isthmus has been lost, and/ or bone defects involving the metaphysis. The loosening of this type is rarely reported in the clinical study. Its occurrence is usually observed in the middle and late stages of prosthesis survival. In our data, only one female patient belongs to the type IV prosthetic loosening. Her long stem rotating hinge keen prosthesis survived for 7 y before the second revision. We initially applied a custom short stem prosthesis in the revision of type IV, resulting in a 1.5 cm postoperative limb shortening. One year after the follow-up, the prosthesis was in a good position, and the MSTS score was 22 (Table 4, Fig. 9). This failure belongs to large-segmental or ultralong bone defects. Revision of this type of defect is challenged by three issues: (1) The bone defect is serious, and the residual bone is too short to support and fix the traditional lengthened stem prosthesis; (2) The defect area is too long, and conventional bone transplantation fails to meet reconstruction needs; (3) Severe limb shortening, and unequal length of both lower limbs were observed in the late stages of the unreserved epiphysis. Therefore, this revision is faced with considerable challenges, and whether to retain the hip joint determines the choice of revision. The main methods applied for the reconstruction of this kind of femoral defect include customized lateral plate locking femur prosthesis [53], 3D-printed short femoral stem prosthesis [37], and total femoral replacement [54].

Based on our retrospective analysis of the case data of 3 treatment centers and combined with previous literature on prosthesis revision, we summarize the revision strategy for the different classifications of bone defects in the revision of aseptic loosening. For type I and II, the original prosthesis can be tried if there is less bone defect, and most prosthesis loosening can be revised by the longer-stem prosthesis. If necessary, the cortical strut allograft can be used to repair the defect or strengthen the fixation. For type III, we recommend the use of a custom short stem prosthesis for the fixation of short residual femoral segments. In addition, telescopic techniques [45] and CPS prostheses [46, 47] reported in the literature are also options. For type IV, we recommend the use of a custom short stem prosthesis combined with a lateral steel plate [53] and screws to preserve the original hip joint structure and function as much as possible.

## Conclusion

In conclusion, the classification of the aseptic loosening of distal femoral prostheses can be used to accurately assess residual bone and bone mass in the proximal femur. Aseptic loosening of the distal femoral prosthesis can be divided into 4 types. The reconstruction methods of our centers for different types of bone defects can offer some reference value in the revision of aseptic loosening.

## Limitation

First, this study is a retrospective descriptive study. Owing to the small size of the sample, no statistical analysis was performed between groups. Moreover, the level of clinical evidence for the proposed treatment strategies is limited. Second, the classification proposed in this study is based on imaging, and the understanding of aseptic loosening is inevitably limited. Finally, although the study included clinical data from 3 bone tumor diagnostic and treatment centers, the accumulated clinical cases remain small. Moreover, some special cases may have imaging findings that cannot be classified into a certain type or subtype. In future research, large sample size is needed to verify the accuracy of this classification.

## Data Availability

The datasets generated and/or analyzed during the current study are not publicly available due to their containing information that could compromise the privacy of research participants but are available from the corresponding author upon reasonable request.

## Abbreviations

MSTS: Musculoskeletal Tumor Society
DFR: Distal femoral replacement
